# Challenges, current innovations, and opportunities for managing type 2 diabetes in frail older adults: a position paper of the European Geriatric Medicine Society (EuGMS)—Special Interest Group in Diabetes

**DOI:** 10.1007/s41999-025-01168-1

**Published:** 2025-02-27

**Authors:** Virginia Boccardi, Gülistan Bahat, Cafer Balci, Isabelle Bourdel-Marchasson, Antoine Christiaens, Lorenzo Maria Donini, Sibel Cavdar, Stefania Maggi, Serdar Özkök, Tajana Pavic, Stany Perkisas, Stefano Volpato, Muhammad Shoaib Zaidi, Andrej Zeyfang, Alan J. Sinclair

**Affiliations:** 1 Division of Gerontology and Geriatrics, Department of Medicine and Surgery, University of Perugia, Piazzale Gambuli 1, 06132 Perugia, Italy; 2 Division of Geriatrics, Department of Internal Medicine, Istanbul Medical Faculty, Istanbul University, Çapa, 34093 Istanbul Turkey; 3 Division of Geriatric Medicine, Department of Internal Medicine, Faculty of Medicine, Hacettepe University, Ankara, Turkey; 4 CNRS, CRMSB, UMR 5536, University of Bordeaux, Bordeaux, France; 5 University Hospital of Bordeaux, Bordeaux, France; 6 Fund for Scientific Research—FNRS, 1000 Brussels, Belgium; 7 Clinical Pharmacy and Pharmacoepidemiology Research Group, Louvain Drug Research Institute (LDRI), Université Catholique de Louvain, 1200 Brussels, Belgium; 8 Department of Experimental Medicine, Sapienza University, Rome, Italy; 9 Division of Geriatrics, Department of Internal Medicine, Izmir City Hospital, Bayraklı, 35540 Izmir Turkey; 10 CNR Institute of Neuroscience, Aging Branch, Padua, Italy; 11 Department of Gastroenterology and Hepatology, University Hospital Center Sestre Milosrdnice, Zagreb, Croatia; 12 University Centre for Geriatrics ZNA (Ziekenhuis Netwerk Antwerpen), University of Antwerp, Antwerp, Belgium; 13 Dipartimento di Scienze Mediche, Università di Ferrara, Ferrara, Italy; 14 Department of Internal Medicine, King Saud University Medical City, Riyadh, Saudi Arabia; 15 Department of Internal Medicine, Geriatric Medicine and Diabetology, Medius Klinik Ostfildern-Ruit, Ostfildern, Germany; 16 Institute of Epidemiology and Medical Biometry, ZIBMT, Ulm University, Ulm, Germany; 17 King’s College London, London, UK

**Keywords:** Aging, Diabetes, Frailty, Geriatrics, Novel, Technology

## Abstract

**Purpose:**

This position paper aims to address the challenges of managing type 2 diabetes mellitus (T2DM) in frail older adults, a diverse and growing demographic with significant variability in health status. The primary research questions are: How can frailty assessment be effectively integrated into diabetes care? What strategies can optimize glycaemic control and outcomes for frail older adults? How can innovative tools and technologies, including artificial intelligence (AI), improve the management of this population?

**Methods:**

The paper uses the 5 I’s framework (Identification, Innovation, Individualization, Integration, Intelligence) to integrate frailty into diabetes care, proposing strategies such as frailty tools, novel therapies, digital technologies, and AI systems. It also examines metabolic heterogeneity, highlighting anorexic-malnourished and sarcopenic-obese phenotypes.

**Results:**

The proposed framework highlights the importance of tailoring glycaemic targets to frailty levels, prioritizing quality of life, and minimizing treatment burden. Strategies such as leveraging AI tools are emphasized for their potential to enhance personalized care. The distinct management needs of the two metabolic phenotypes are outlined, with specific recommendations for each group.

**Conclusion:**

This paper calls for a holistic, patient-centered approach to diabetes care for frail older adults, ensuring equity in access to innovations and prioritizing quality of life. It highlights the need for research to fill evidence gaps, refine therapies, and improve healthcare integration for better outcomes in this vulnerable group.

**Graphical abstract:**

## Key summary points

**Aim:**

To explore the challenges and opportunities in managing type 2 diabetes mellitus (T2DM) in frail older adults and develop tailored, patient-centered strategies to improve outcomes by integrating frailty into routine diabetes care.

**Findings:**

Frailty complicates diabetes management, creating a need for individualized approaches based on the extremes of metabolic phenotypes, namely, the anorexic malnourished and sarcopenic obese. Innovations in pharmacological treatments, digital tools like continuous glucose monitoring (CGM), and AI-driven solutions hold promise for optimizing care. Simplified regimens, relaxed glycemic targets, and holistic strategies are essential for addressing the complexities of this population.

**Message:**

A multidisciplinary, individualized approach that incorporates frailty assessment, innovative therapies, and technology can enhance the management and quality of life for frail older adults with T2DM.

## Graphical abstract

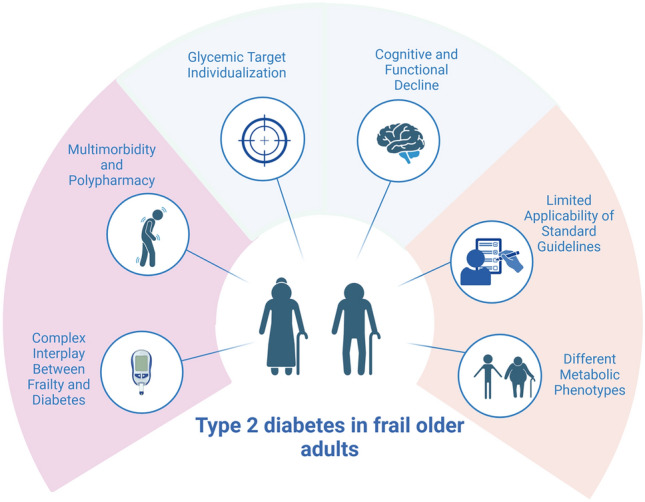

## Introduction

Type 2 diabetes (T2DM) is a prevalent health condition, currently impacting 10.5% of the adult population in Europe. Projections suggest that its prevalence will rise to 13% by 2045 partially due to aging populations and increasing obesity rates [1]. Approximately 25% of adults aged 65 and older have diabetes (from the Global Burden of Disease current dataset of the Institute of Health Metrics, Seattle), with the global prevalence expected to rise due to factors like sedentary lifestyles and poor dietary habits [2]. An estimated 74 million adults (11.9% of men and 10.9% of women) are living with diabetes in the World Health Organization (WHO) European Region [3]. Since 2000, approximatively 50% of people receiving treatment for diabetes in Europe are older than 65 or 70 years and 25% older than 75 years [4]. Type 2 diabetes mellitus (T2DM) is the predominant form, accounting for about 90–95% of all diabetes cases. Again, advancements in diabetes care and improved management of complications have contributed to enhanced life expectancy for a growing number of older individuals living also with Type 1 diabetes mellitus (T1DM) [5]. A recent study reported that globally, the prevalence of T1DM among individuals aged 65 and older increased by 180% between 1990 and 2019, rising from 1.3 to 3.7 million cases [5].

Older adults, typically defined as individuals aged 65 and above, represent a diverse demographic with varying health statuses, from those who are healthy and independent to those with chronic illnesses and disabilities [6, 7]. While many maintain high levels of physical and cognitive function, they still require age-related preventive care and monitoring for common chronic diseases [7]. Some older adults face more complex health issues due to the comorbidities, physiological, cognitive, and functional changes associated with aging [8]. Geriatric care requires a comprehensive approach that addresses the management of multiple chronic diseases, polypharmacy, and geriatric syndromes, while also considering the social and psychological dimensions of aging. These factors are strongly linked to frailty, as they can exacerbate vulnerability and reduce resilience, underlying the need for tailored, multidimensional interventions to optimize outcomes in older adults. Frailty is defined as a state of increased vulnerability to psychological and physical stressors because of a reduction in physiological reserve, which limits the capacity to maintain homeostasis [9]. This vulnerable state is independent of age but more common with aging. In fact, the prevalence of frailty reaches around 25% in people aged ≥ 85 years and affects about 32–48% of older people with diabetes [10]. Diabetic patients are more likely to be frail than non-diabetic older adults—a condition associated with many adverse outcomes, such as disability, hospitalizations, and increased mortality [11].

The relationship between frailty and diabetes is complex and potentially bi-directional [10]. T2DM is associated with increased frailty risk, and a longitudinal study indicates that it predicts transitions to higher frailty levels [12]. It remains unclear whether frailty directly contributes to the development of diabetes. While frailty is recognized as an independent risk factor for morbidity and mortality in diabetes [13], its role in determining HbA1c levels or influencing diabetes management is not fully understood. Most importantly, frailty induces body composition changes that influence the metabolic state and affect diabetes trajectory [14]. Interestingly, frailty includes a broad metabolic spectrum, manifesting in distinct phenotypes that highlight its complexity and clinical implications [15, 16]. One such phenotype is “sarcopenic obesity”, characterized by significant muscle mass loss accompanied by increased visceral fat [17]. This phenotype is associated with heightened insulin resistance and an amplified cardiovascular risk, which exceeds the risk conferred by obesity or sarcopenia alone [18]. In individuals with sarcopenic obesity [19], the progression of diabetes is accelerated, necessitating more intensive hypoglycaemic therapy alongside a proactive approach to cardiovascular risk reduction. Conversely, the “anorexic malnourished” phenotype is defined by substantial weight loss and decreased insulin resistance [16]. In these individuals, the trajectory of diabetes is typically slowed, shifting the therapeutic focus toward de-intensifying hypoglycaemic regimens. Management in this phenotype prioritizes symptom control and improving quality of life, rather than stringent metabolic targets. These contrasting phenotypes underline the importance of individualized care in frail older adults with diabetes, emphasizing tailored interventions that address both metabolic and functional needs, independent of age [20].

Tailored healthcare strategies are needed to address both diabetes and frailty, including individualized glycaemic targets, functional assessments, and preventive measures [21]. Considering such evidence, diabetes care must be individualized due to the highly heterogeneous nature of this population [22, 23]. Treatment intensity [24], glycaemic targets, and therapy choices should be tailored based on the patient’s functional status, life expectancy, nutritional status, metabolic profile, and preferences [25]. A comprehensive geriatric assessment (CGA) that evaluates medical history, functional and cognitive status, nutritional needs, geriatric syndromes, and psychosocial factors is essential for creating personalized care plans. Table 1 outlines the factors influencing suboptimal diabetes care in older and vulnerable individuals and provides strategies for improvement, highlighting the multifactorial nature of diabetes management in this population.

**Table 1:** Factors influencing suboptimal diabetes care in older adults and strategies for improvement

Factors	Strategies
Heterogeneity of older adults	Implement individualized care plans based on frailty levels, functional status, life expectancy, comorbidities, and patient preferences
Different metabolic phenotypes	The spectrum includes two key metabolic phenotypes in frail older adults with diabetes: “anorexic malnourished” (AM), marked by undernutrition and muscle loss, and “sarcopenic obese” (SO), characterized by excess fat and reduced muscle mass. These extremes underscore the need for tailored management strategies
Risk of hypoglycaemia	Adjust glycaemic targets (e.g., HbA1c 7.5–8.0% for frail individuals) and avoid medications with high hypoglycaemia risks, such as sulfonylureas
Cognitive decline and physical impairments	Simplify treatment regimens, involve caregivers in care plans, and use long-acting medications or devices to reduce the complexity of diabetes self-management
Polypharmacy and medication interactions	Regularly review medication lists to minimize non-essential drugs, reduce interactions, and optimize medication regimens tailored to the patient’s needs
Comorbidities	Integrate diabetes management with care for other chronic conditions, prioritizing medications with dual benefits (e.g., SGLT2 inhibitors for heart failure or nephropathy)
Limited access to multidisciplinary care	Enhance access to geriatric care teams, including endocrinologists, dietitians, and social workers, to provide comprehensive and coordinated care
Inadequate patient and caregiver education	Empower patients and caregivers through education about self-management, recognizing symptoms of complications, and optimizing lifestyle interventions
Healthcare system barriers	Foster integrated care models and implement telemedicine or community-based services to improve access and continuity of care
Socioeconomic challenges	Advocate for affordable medications, provide social support programs, and address social determinants of health to improve adherence and outcomes
Inadequate focus on preventive care	Prioritize vaccination, routine screening (e.g., foot and eye exams), for geriatric syndromes and early intervention to reduce the risk of complications and hospitalizations

*HbA1c* hemoglobin A1c, *SGLT2* sodium-glucose cotransporter-2 inhibitors

## Objectives

**Fig. 1:**
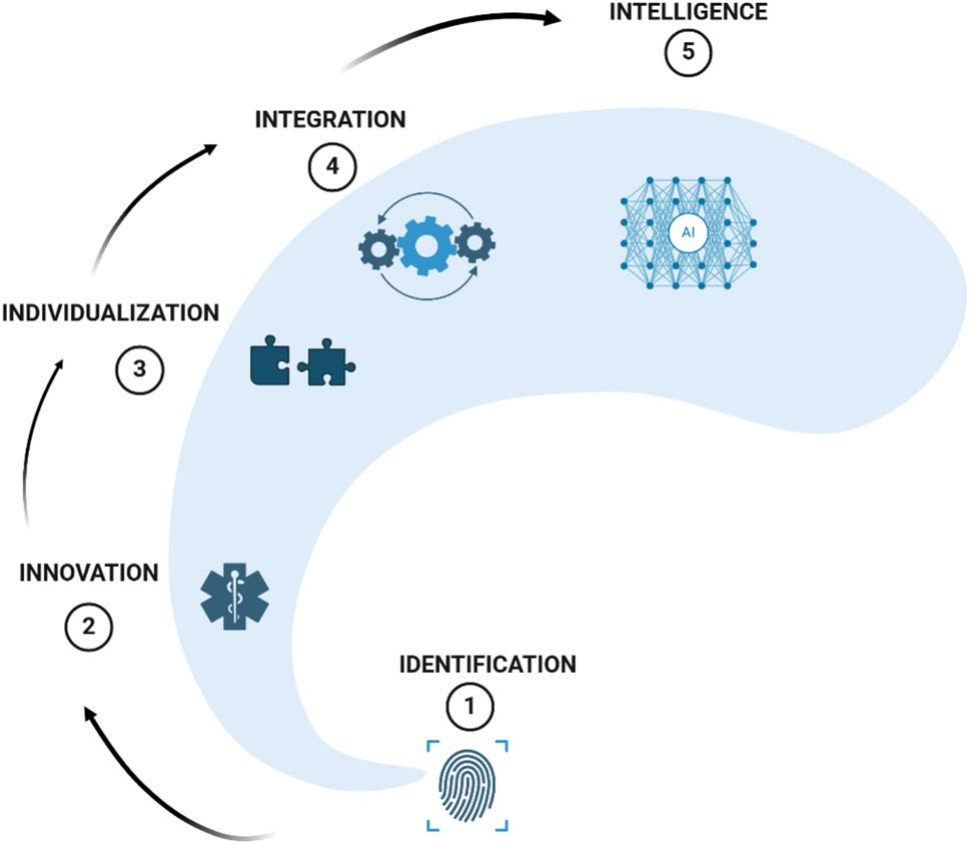
The five “I” pillars of advancing diabetes care in frail older adults: Identification, Innovation, Individualization, Integration, and Intelligence. The figure illustrates the five critical pillars of diabetes management advancements in frail adults with T2DM: (1) Identification of frailty, (2) Innovation in pharmacological treatments, (3) Individualization of care tailored to patient-specific needs, (4) Integration of digital health technologies to optimize management, and (5) Intelligence through AI-driven solutions for personalized and efficient care. (Created with BioRender)

**Fig. 2:**
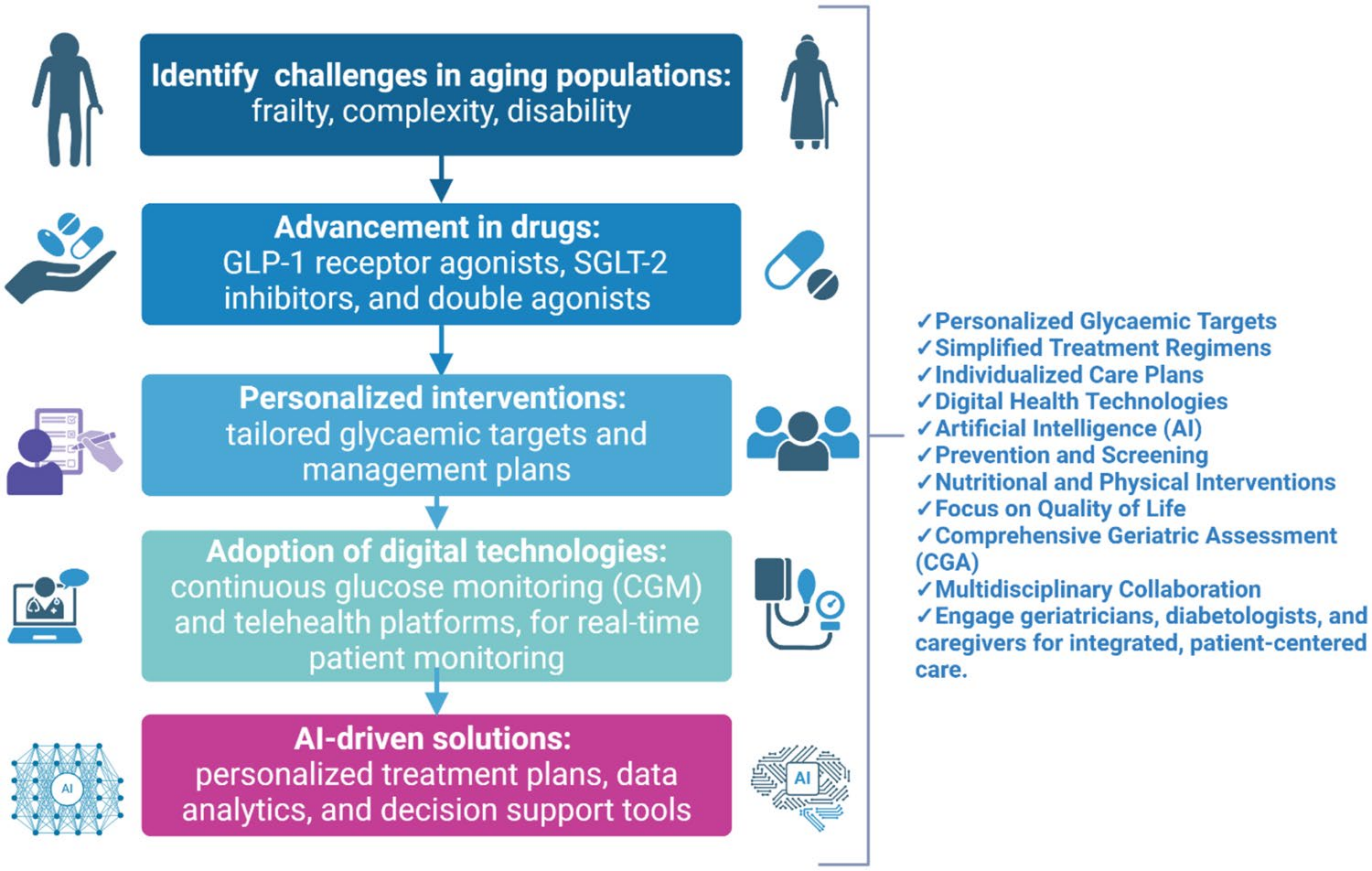
Comprehensive diabetes management in frail older adults. Comprehensive diabetes management in frail older adults integrates key steps to address the unique challenges of aging populations. The framework starts with identifying frailty, complexity, and disability, followed by advancements in pharmacological treatments like GLP-1 receptor agonists, SGLT2 inhibitors, and double agonists. Personalized interventions focus on tailored glycaemic targets and care plans. The adoption of digital technologies, such as continuous glucose monitoring (CGM) and telehealth platforms, enhances real-time patient monitoring. Finally, AI-driven solutions enable personalized treatment plans, advanced data analytics, and decision support tools, ensuring a holistic and patient-centered approach to care. (Created with BioRender)

This position paper has been developed by the Diabetes Special Interest Group of the European Geriatric Medicine Society, reflecting a collaborative effort by experts in geriatric care and diabetes management. This work aims to explore the current challenges and emerging opportunities in the management of T2DM in frail populations, a demographic that presents unique clinical challenges due to its complexity. The demographic reality that the 80 + population, often referred to as the “oldest old,” represents the fastest-growing segment globally [26]. With frailty increasing as individuals age, this demographic shift will result in a growing number of frail older adults requiring comprehensive medical care, including diabetes management [27]. Specifically, this paper addresses the “vulnerable” subset of the aging population—individuals who are not only living with T2DM but also contend with additional health issues, such as multiple comorbidities and functional limitations, independent of chronological age. These factors complicate diabetes management and increase the risk of adverse outcomes, requiring more individualized care approaches. This position paper advances the EuGMS-EDWPOP (European Geriatric Medicine Society-European Diabetes Working Party for Older People) primary care guideline [4] by addressing critical gaps and focusing on frail older adults with T2DM, and integrating novel pharmacological treatments, digital health innovations, and artificial intelligence (AI). It introduces new therapies such as double agonists like tirzepatide and ultra-long-acting insulins, emphasizing their potential benefits while addressing unique challenges like muscle preservation in frailty. Furthermore, it expands significantly on digital tools, discussing AI-driven personalized treatment plans, continuous glucose monitoring (CGM), and telehealth, which enhance patient engagement and care quality. The paper is structured around five critical areas of diabetes management (the five “I”) that have undergone remarkable advancements in recent years (Fig. 1), which hold significant promise for improving outcomes in older adults with T2DM: (1) Identification of frailty, (2) Innovation in drugs, (3) Individualization of treatment, (4) Integration of technology, and (5) Intelligence through AI. By focusing on these areas, the paper aims to provide a comprehensive overview of how T2DM management can be optimized through novel drug therapies, personalized treatment regimens, and the incorporation of technological innovations, including artificial intelligence, to enhance clinical decision-making and patient care. Following this approach, a framework for comprehensive diabetes management in frail older adults is presented in Fig. 2.

## Five I’s in the management of T2DM in frail older adults

Advancements in diabetes management, particularly with new drug therapies and treatment strategies, represent a significant revolution in the field. However, it is essential to recognize that much of the current evidence supporting these innovations has not been thoroughly assessed in frail older adults. Over the past decade, several novel drug classes have been introduced for diabetes management, offering more options for vulnerable population [28]. However, it is essential to recognize that much of the current evidence supporting these innovations has not been thoroughly assessed in vulnerable populations, including frail older adults. While the existing studies are reassuring, they remain few and often exclude the very old individuals who would benefit most from these advancements—those with multiple comorbidities, functional impairments, and frailty. This gap in research presents a crucial opportunity for future studies to include real-world data from frail older adults, particularly those who are underrepresented in clinical trials. By addressing this gap, we can ensure that these innovations are truly transformative and accessible for vulnerable older populations. These newer therapies are often more effective in reducing glycaemic variability and offer benefits beyond glycaemic control [29]. In this context, frailty identification plays a critical role.

### “Identification” of frailty beyond age

Frailty identification is essential for optimizing care in older adults, as it helps avoid overtreatment, reduces the risk of adverse outcomes, and guides interventions to maintain functional independence and quality of life. Frail individuals have significantly reduced resilience and are more vulnerable to acute stressors and less able to recover from illnesses. The key differences between frail patients and healthier older adults are in health complexity: frail individuals often have multiple comorbidities, polypharmacy, and cognitive or functional impairments [30]. Frailty identification could be used as surrogate indicators of biological aging, as they reflect the rate of health and functional decline over time. Independent of chronological age, its presence has been strongly linked to adverse outcomes such as hospitalization, disability, and mortality [31, 32]. Moreover, it is important to recognize that frailty is a dynamic condition, presenting opportunities for targeted interventions to mitigate its effects and even achieve improvements [33]. Thus, the integration and recognition of the concept of frailty in clinical practice is an urgent need, particularly for general practitioners managing T2DM.

Despite the lack of a universal consensus on how to best assess frailty, the most widely used and validated frailty models are the physical frailty phenotype [33] and the frailty index (FI; multidimensional deficit accumulation) [34]. The Fried Frailty Phenotype [33] provides a detailed assessment, including five criteria: unintentional weight loss, exhaustion, physical activity, walking speed, and grip strength. The FI assesses instead the accumulation of health deficits with aging across a range of domains of functioning (physical, psychological, and social) [34]. Nevertheless, these measures are time- and resource-consuming, which limits their incorporation into routine clinical practice [35]. One of the most frequently adopted frailty measure in clinical settings is the Clinical Frailty Scale (CFS) developed by Rockwood et al. [36]. This global measure of fitness and frailty can be completed in less than 5 min, requiring no specialized equipment. To address the need for a quicker yet reliable assessment, the Simplified Fried Frailty Scale has been developed and clinically validated [37]. This adaptation simplifies the original Fried criteria into a format that can be completed in less than 5 min without the need for specialized equipment. Automated frailty scores, based on routinely collected electronic health records (EHRs) or administrative claims data, have also recently been developed which includes the electronic frailty index (eFI) [38]. Routine frailty screening can help planning of resource allocation and identifying patients who would benefit most from a CGA. By incorporating these validated tools in clinical practice, healthcare professionals can enhance the quality of care for older adults, improving outcomes and aligning treatment with the patient’s overall health status and goals. Table 2 reports a simplified workflow for frailty assessment in primary care. The table provides a step-by-step workflow to assess frailty, ensuring efficient evaluation and management tailored to older adults’ needs.

**Table 2:** Simplified workflow for frailty assessment in primary care beyond chronological age

Step	Actions	Tools/details
Step 1: Identify older adults at risk	Screen older adults aged ≥ 65 years with multiple comorbidities, recent weight loss, functional decline, or falls	Quick screening questions: “Have you experienced unintended weight loss in the past 6 months” “Do you feel fatigued or lack energy most days?” “Do you have difficulty walking a short distance (e.g., 400 m)?”
Step 2: Select an appropriate frailty assessment tool	Use validated tools to assess frailty. Select based on time availability and resources	**Clinical Frailty Scale (CFS):** < 5 min, no equipment, scores ≥ 5/8 indicate frailty
		**Full Fried Frailty Phenotype:** 5–10 min, includes grip strength (dynamometer required) and walk speed
		**Simplified Fried Frailty Scale:** < 5 min, no equipment, evaluates weight loss, exhaustion, physical activity, walking speed
		**Frailty Index (FI):** Comprehensive tool based on accumulation of health deficits (e.g., symptoms, comorbidities, disabilities)
		**Electronic Frailty Index (eFI):** Calculated using routine electronic health record data, providing an objective measure of frailty based on accumulated deficits. The eFI calculates a frailty score by dividing the number of deficits present by the total possible: uses 36 validated deficits. The eFI score ranges from 0 to 1, with higher scores indicating greater frailty
Step 3: Stratify frailty severity	Categorize patients based on frailty assessment results	**Non-frail (Fit):** Robust health, no frailty markers
		**Pre-frail:** 1–2 frailty markers
		**Frail:** ≥ 3 markers or CFS score ≥ 5
Step 4: Conduct Comprehensive Geriatric Assessment (CGA)	Evaluate medical, functional, and psychosocial aspects for frail or pre-frail individuals	Physical function: i.e., gait speed, grip strength. Nutritional status: i.e., Mini Nutritional Assessment. Cognitive function: i.e., Mini Mental State Examination. Polypharmacy review: i.e., deprescribe unnecessary medications. Psychosocial needs: i.e., caregiver support, living conditions
Step 5: Develop an individualized management plan	Create a personalized care plan based on frailty status and functional needs	**Functional goals:** Maintain independence, mobility, and quality of life. **Interventions:** Prescribe physical therapy, resistance training, and nutritional support. **Glycaemic targets:** Adjust based on frailty severity (e.g., HbA1c: 7.5–8.5% for frail individuals). **Monitoring:** Schedule regular follow-ups to reassess functional and clinical status

### “Innovation” in pharmacological treatments

**Fig. 3:**
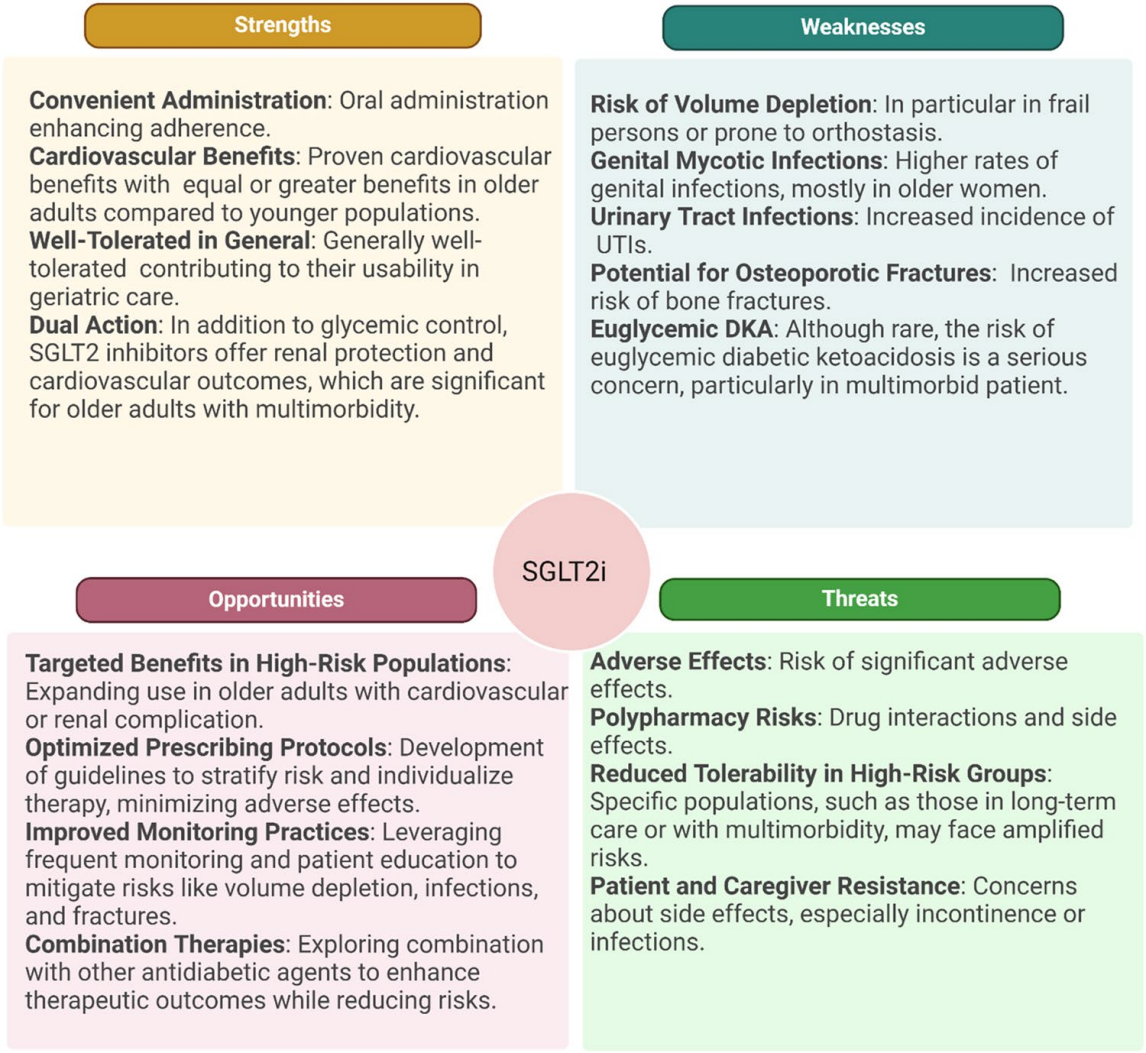
SWOT analysis (Strengths, Weaknesses, Opportunities, Threats) regarding the use of SGLT2 inhibitors (SGLT2i) in frail older adults. *DKA* diabetic ketoacidosis, *UTI* urinary tract infection. (Created with BioRender)

Recent innovations in pharmacological treatments offer significant opportunities for managing diabetes in frail patients, providing benefits beyond glycaemic control, such as cardiovascular, renal, and neuroprotective effects. Advanced therapies open new possibilities for addressing complex cases in this vulnerable population. However, it is crucial to carefully balance the benefits and risks, including potential impacts on muscle mass and nutritional status. The primary drug developments include SGLT2i (Sodium-Glucose Cotransporter-2 Inhibitors), GLP-1 RA (Glucagon-Like Peptide-1 Receptor Agonists), and double agonists (tirzepatide—dual glucose-dependent insulinotropic polypeptide (GIP) and GLP-1 RA). In highly multimorbid individuals, reducing postprandial blood glucose is crucial for improving daily living and simplifying treatment while minimizing hypoglycaemia risk [39]. This benefit is evident even in early stages of therapy with SGLT2i, which offer effective glycaemic control making them ideal for managing complex cases. SGLT2inot only reduce blood glucose levels but also provide cardiovascular and renal protection [40]. Moreover, recent evidence shows that they offer protective effects on several neurodegenerative diseases such as Parkinson’s diseases and dementia [41]. These benefits make SGLT2i particularly valuable for older adults with comorbid heart failure, chronic kidney disease, and/or neurodegenerative risk [42] or diseases [43]. SGLT2i , such as empagliflozin and dapagliflozin, have been shown to reduce heart failure hospitalizations [44, 45] and slow the progression of diabetic nephropathy, which is highly relevant in frail populations [46, 47]. SGLT2i represent the first drug class to significantly reduce mortality and improve outcomes in patients with heart failure with preserved ejection fraction (HFpEF, also known as diastolic dysfunction) [48]. They also reduce cardiovascular outcomes like myocardial infarction, stroke, and revascularisation (with canagliflozin) [49]. This is true in persons living with diabetes but also without leading to guidelines extending their use largely [50]. However, in clinical practice, careful consideration is required when prescribing these medications, especially for frail patients with conditions, such as malnutrition, orthostatic hypotension, a high risk of falls, osteoporotic fractures, recurrent urinary tract infections, or urinary incontinence [51, 52]. It is also essential to inform patients about the potential drawbacks and risks associated with these drugs to ensure informed decision-making and optimal care. Figure 3 shows an SWOT analysis (Strengths, Weaknesses, Opportunities, Threats) based on Standards of Care in Diabetes (2025) [53] regarding the use of SGLT2i in frail older adults.

**Fig. 4:**
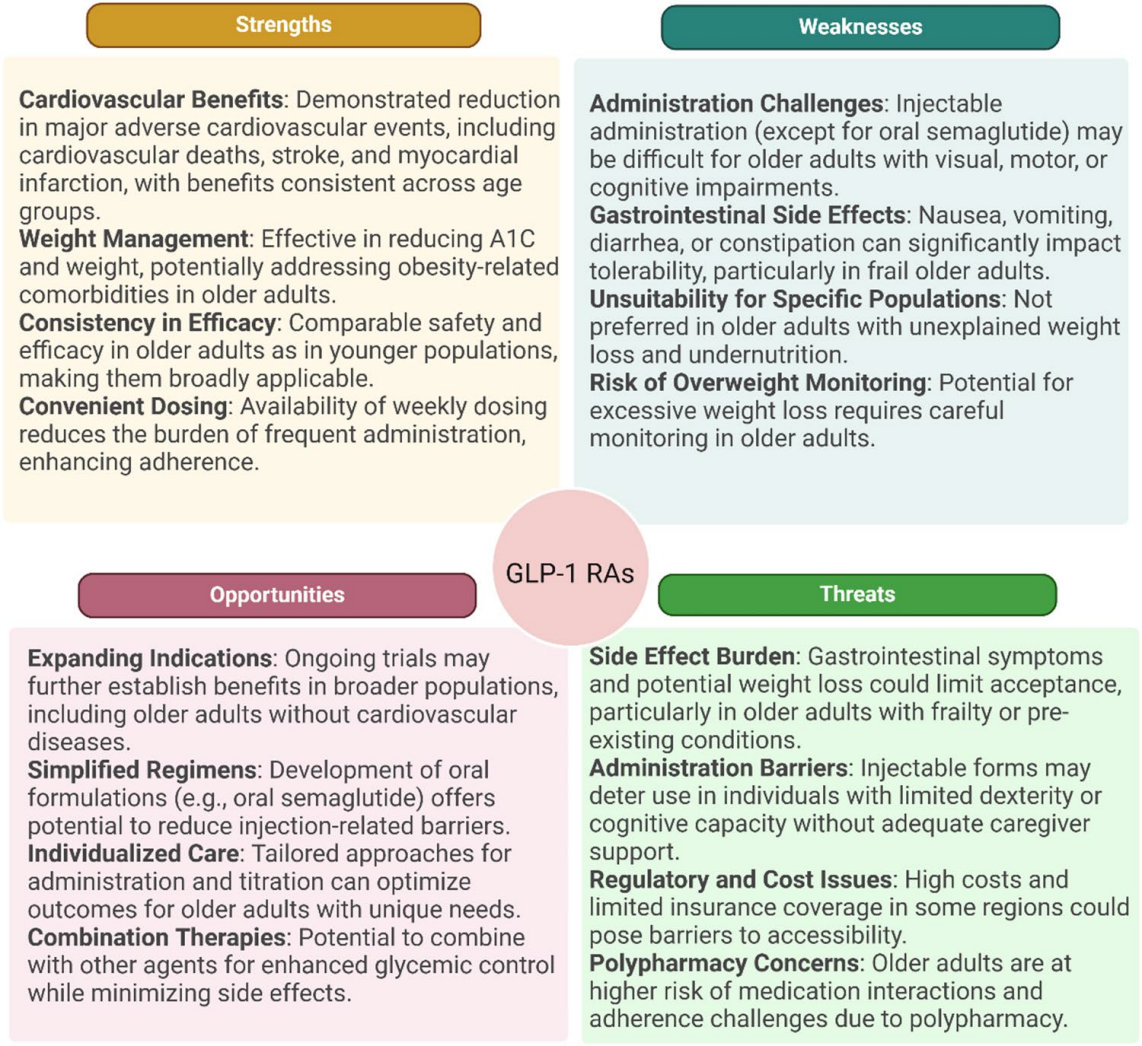
SWOT analysis (Strengths, Weaknesses, Opportunities, Threats) regarding the use of GLP-1 RA in frail older adults. (Created with BioRender)

GLP-1 RA, such as Liraglutide and Semaglutide, improve glycaemic control by enhancing insulin secretion and reducing appetite, leading to weight loss [54, 55]. These drugs also offer cardiovascular benefits and reduce the risk of major adverse cardiovascular events in high-risk patients, which is critical given the increased prevalence of cardiovascular disease in older adults with diabetes [56]. The effect in preventing cognitive troubles [57] is possible according to several post hoc analysis of clinical trials and was also observed in real life [56]. Double agonists, such as tirzepatide, represent a novel class of antidiabetic drugs that target both the GLP-1 (glucagon-like peptide-1) and GIP (glucose-dependent insulinotropic polypeptide) receptors. By simultaneously activating these pathways, tirzepatide enhances insulin secretion, suppresses glucagon release, and reduces appetite, leading to improved glycaemic control and significant weight loss [58]. This dual mechanism makes tirzepatide a promising option for managing type 2 diabetes, particularly in patients with obesity. Recent clinical trials have demonstrated its superior efficacy on glycaemic control and weight management compared to traditional GLP-1 RA, marking it as a key development in diabetes care [59, 60]. Overall, despite their promising benefits, these innovative therapies have limitations in frail individuals, particularly regarding their impact on muscle mass. While these medications effectively promote weight loss by reducing fat mass, they may also lead to unintended reductions in lean body mass, including muscle tissue. It was reported that up to 20–50% of weight loss seen with SGLT2i and GLP-1 RA is attributable to loss of lean mass [61]. This is especially concerning in frail older adults, who often have diminished physiological reserves and are already at risk of sarcopenia. Thus, before considering antidiabetic agents with weight-losing property (i.e., metformin, SGLT2i, GLP-1 RA, and tirzepatide) for their glycemic or cardiorenal benefits, we should assess the nutritional status and metabolic phenotype of the individuals and avoid using them in patients with malnutrition or malnutrition risk, at least until their nutritional status is optimized [48, 62]. These agents can cause an already malnourished individual to face a list of hazardous outcomes related to worsening of malnutrition (i.e., increased risk of sarcopenia, frailty, disabilities, hospitalisations, and eventually, mortality). In fact, major guidelines and consensus papers do not recommend weight loss as a primary goal in older adults, whether they are vulnerable or healthy, because patients with diabetes already have an increased risk of malnutrition and malnutrition risk, compared to their counterparts. Therefore, risks and benefits should be thoroughly assessed, and appropriate patients should be wisely selected [63]. Figure 4 shows an SWOT analysis based on Standards of Care in Diabetes (2025) [53] regarding the use of GLP-1RA in frail older adults. Importantly, when using weight-losing antidiabetic agents for weight management, weight loss should not exceed more than 1 kg/week, and this regime should be supported with at least 1 g/kg/day high-quality protein intake (with considering renal functional status) and individualized exercise programs (primarily resistance training [64]), to preserve muscle mass and functions [48]. The net effect of weight loss seen with these medications can be translated in favor of muscle by first and foremost, routine and reliable assessment of muscle health during the treatment, which can be supplied using proper adjustment techniques for muscle mass in especially obese individuals to avoid misdiagnoses, providing sufficient amount of protein and calorie intake, and prescribing individualized resistance exercises [65, 66].

Indeed, in addition to weekly insulin formulations (i.e., icodec), another novel advancement coming in diabetes management for frail patients is the development of long-acting basal insulin analogs with extended duration of action [67]. These newer long-acting insulins, including ultra-long-acting formulations, offer the potential for even greater convenience and improved adherence for patients who struggle with daily or weekly injections [68]. For frail older adults, particularly those with cognitive impairments or limited physical capabilities, the ability to reduce the frequency of injections can significantly ease the burden of diabetes management. The extended duration of action of these insulin products provides a more stable and sustained release of insulin, allowing for better glycaemic control over extended periods without the need for frequent dosing adjustments. This can help to mitigate fluctuations in blood glucose levels and reduce the risks of both hyperglycaemia and hypoglycaemia, which are especially dangerous in frail populations. Moreover, these long-acting insulins could enhance overall quality of life by simplifying treatment regimens and reducing the time and effort required for self-management. Indeed, the higher costs associated with these new-generation therapies can be considered a significant drawback, as they may pose a barrier for patients facing financial challenges. Furthermore, the use of injectable medications often necessitates social support, particularly for patients with cognitive or visual impairments, as well as those with functional limitations [48]. However, as with any new therapy, more research is needed to evaluate the efficacy, safety, and long-term outcomes of these ultra-long-acting insulins in older, frail patients to ensure that they are tailored to the specific needs of this vulnerable population.

### “Individualization” of care tailored to patient-specific needs

#### Comprehensive, person-centered risk–benefit strategies

Managing diabetes in frail older adults requires a person-centered approach that carefully balances the benefits of treatment against potential risks [69]. CGA [70] plays a pivotal role, allowing clinicians to evaluate functional status, mobility, cognitive ability, and psychosocial factors, such as caregiving support and the patient’s capacity to adhere to treatment. Regular reassessment is essential, as changes in health or functional status may require adjustments to treatment plans and targets [71].

The risk of hypoglycaemia is particularly concerning in frail older adults, as it can lead to severe consequences, such as falls, fractures, and hospitalization [16]. Guidelines increasingly emphasize the need for individualized glycaemic targets that prioritize safety and quality of life over strict glycaemic control [72]. Several classification systems have been developed to address the heterogeneity of benefit–risk balance in older adults with T2DM. Among the most widely used is the “3-tiered” system proposed by Blaum and Cigolle, which categorizes older adults into robust, intermediate (or pre-frail), and frail groups. This classification uses functional status, comorbidity burden, and cognitive impairment to stratify patients, guiding treatment goals and intensities [73]. A similar approach has been also previously proposed in managing glucose control in older persons [74]. Again, the more recent system developed by Huang and colleagues employs Latent Class Analysis (LCA) to identify subgroups of older adults with T2DM based on multimorbidity patterns and health trajectories [75]. LCA identifies hidden patterns in data to classify individuals into similar groups. For example, it might reveal subgroups with mostly cardiovascular complications or those experiencing rapid health decline. This approach helps tailor care strategies, allowing healthcare providers to address the unique needs of each subgroup and improve outcomes. Huang’s approach captures the complexity of comorbid conditions and their interplay with diabetes outcomes, offering a nuanced tool for clinical decision-making. However, both classifications have limitations. Blaum and Cigolle’s 3-tiered system, while practical, may oversimplify the diversity within each category, potentially overlooking critical individual variations. Huang’s LCA-based framework is more comprehensive but relies heavily on data availability and advanced analytics, limiting its direct application in routine clinical practice. Emerging precision medicine approaches offer additional potential for tailoring treatment, incorporating genetic, biochemical, and phenotypic data to create highly personalized care strategies. However, these methods face significant barriers in older adults with T2DM [76, 77].

#### Individualized targets for glucose control

The historic emphasis on strict glycaemic control (e.g., HbA1c < 7%) has evolved in recognition of the diverse health profiles among older adults [76]. Individualized HbA1c targets are now recommended, reflecting variations in functional independence, frailty, and life expectancy [77]. While discrepancies remain between existing guidelines regarding specific HbA1c targets, the EuGMS, in collaboration with the EDWPOP [4], has proposed stratified targets based on functional and frailty status (while prefrailty represents an intermediate stage, offering a critical window for intervention), highlighting the need for flexible approaches to glycaemic management and the importance of regular reassessment.

The EuGMS guidelines propose three main categories for target setting:

1. Functionally independent: These patients can manage their diabetes effectively, with an HbA1c target of 7.0–7.5% (53–58.5 mmol/mol) considered appropriate for achieving meaningful glycaemic control without excessive risk. Moreover, if the patient is on a medication with no risk of hypoglycaemia (e.g., metformin, SGLT2i, or DPP-4 inhibitors), we propose adopting a flexible target of up to 6–6.5%.
2. Partially dependent and pre-frail: For individuals with some functional limitations but moderate independence, a target of 7.5–8.0% (58.5–63.9 mmol/mol) balances glycaemic control with safety, minimizing the risk of hypoglycaemia.
3. Frail or dependent: Patients with significant functional impairments, frailty, or limited life expectancy benefit from less stringent targets of 8.0–8.5% (63.9–69.4 mmol/mol), prioritizing safety and quality of life over tight control.

These targets recognize that the risks of overtreatment, including hypoglycaemia and treatment burden, often outweigh the benefits of aggressive glycaemic control in older adults with complex health profiles. The overarching goal is to tailor glycaemic targets to the individual’s health status, ensuring a focus on independence, safety, and well-being [25]. Accordingly, the American Diabetes Association (ADA) 2025 Guidelines [78] recommend against relying on HbA1c as a primary metric in patients with very poor or complex health conditions. Given the minimal benefits of stringent glycaemic targets in these individuals, the guidelines emphasize preventing hypoglycaemia and symptomatic hyperglycaemia while prioritizing the enhancement of quality of life. This approach is particularly important for patients with extreme frailty, terminal illnesses, or limited life expectancy. In addition to focusing on HbA1c targets, it is essential to emphasize the importance of limiting glycaemic variability, a key concept in diabetes care. Glycaemic variability refers to fluctuations between hypo- and hyperglycaemic phases, which can occur even when HbA1c levels are within the desired range [36, 79]. These fluctuations are clinically significant as they are associated with an increased risk of both acute complications (e.g., hypoglycaemia) and long-term adverse outcomes, including cardiovascular events and oxidative stress. Addressing glycaemic variability involves not just achieving average glucose targets but also stabilizing blood glucose levels throughout the day.

#### De-intensification of glucose-lowering medications

De-intensification of treatment is increasingly recognized as an essential strategy for managing diabetes in older adults, particularly those with frailty, multiple comorbidities, or limited life expectancy [80]. Regular medication reviews are crucial to identify unnecessary or potentially harmful treatments. Deprescribing, when undertaken systematically, can reduce polypharmacy, lower the risk of adverse drug interactions, and improve treatment adherence.

Simplification of the treatment regimen further supports patient safety and quality of life. In patients aged 80 years or older, HbA1c levels below 6.5%, or under 7% in specific circumstances, are generally not recommended. This applies particularly to individuals experiencing frailty, those in the end-of-life phase, patients with frequent hypoglycaemic episodes, those on complex treatment regimens, or individuals with impaired kidney function, dementia, moderate-to-severe frailty, or residing in long-term care (LTC) facilities [74]. Switching to long-acting insulin analogs, once-daily oral medications, or agents with lower hypoglycaemic risk, such as metformin, SGLT2i or GLP-1 RA, can help reduce the complexity of diabetes management. Dipeptidyl peptidase-4 (DPP-4) inhibitors are not a new class of oral antidiabetic medications; they enhance incretin hormone activity, thereby improving glucose homeostasis. However, DPP-4 inhibitors are suitable for use across the full spectrum of frailty, including individuals who are anorexic-malnourished or sarcopenic-obese [48]. This is due to their weight-neutral properties, low risk of hypoglycaemia, and favorable safety profile, making them an adaptable option for diverse patient populations. While these drugs are relatively weak and do not significantly impact major cardiovascular outcomes, age-stratified analyses of cardiovascular trials may lack the power to detect differences. DPP-4 inhibitors are particularly useful for older adults with mild hyperglycaemia, a high risk of hypoglycaemia, or contraindications to metformin [53]. In this context, deprescribing is not merely about reducing the number of medications but rather a strategic approach to optimize safety and efficacy while addressing the patient’s evolving needs [81]. Despite the many promises offered by de-intensification and simplification of glucose-lowering drugs, evidence is not yet available. Efforts should be made to support future-related recommendations [80].

#### Individualization of non-glycaemic management

Effective management of diabetes in older adults extends beyond glycaemic control to address broader health needs and age-related changes. Nutritional management is a cornerstone of care, particularly given the prevalence of sarcopenia and sarcopenic obesity in this population [82]. Nutritional strategies [83] should prioritize maintaining muscle mass and functional capacity through adequate protein intake while avoiding rapid weight loss, especially in patients receiving GLP-1 RA [84]. These interventions are essential to mitigate frailty, enhance physical performance, and improve overall health. Preventive care also plays a crucial role in this population, but it should be differentiated based on the degree of frailty and life expectancy. For all older adults, including the frailest, vaccination remains a cornerstone of preventive care. Vaccines against influenza, pneumococcus, zoster, respiratory syncytial virus, and COVID-19 significantly reduce the risk of infection-related complications, which can be devastating in frail individuals [85]. However, in the frailest individuals with very limited life expectancy, certain preventive strategies, such as routine screening for diabetes-related complications (e.g., retinopathy or foot ulcers), may be burdensome and provide limited benefit [86]. In contrast, for those with greater functional reserve, these screenings are crucial to preventing long-term disability and maintaining independence [87]. However, vaccine hesitancy is a phenomenon already known in diabetics that needs solutions.

Cardiovascular risk management through blood pressure control, lipid regulation, and the promotion of heart-healthy behaviors is similarly vital, given the high incidence of cardiovascular events in older adults. At least annual screening of geriatric syndromes (e.g., cognitive impairment, depression, urinary incontinence, falls, chronic pain, and frailty) is recommended to improve diabetes management and quality of life [78]. Addressing psychosocial factors, such as caregiver support and patient preferences, further enhances the individualization of care. Shared decision-making ensures that treatment plans align with the patient’s goals and values, fostering trust and improving adherence.

### “Integration” of digital health technologies and “intelligence” through AI-driven solutions

**Fig. 5:**
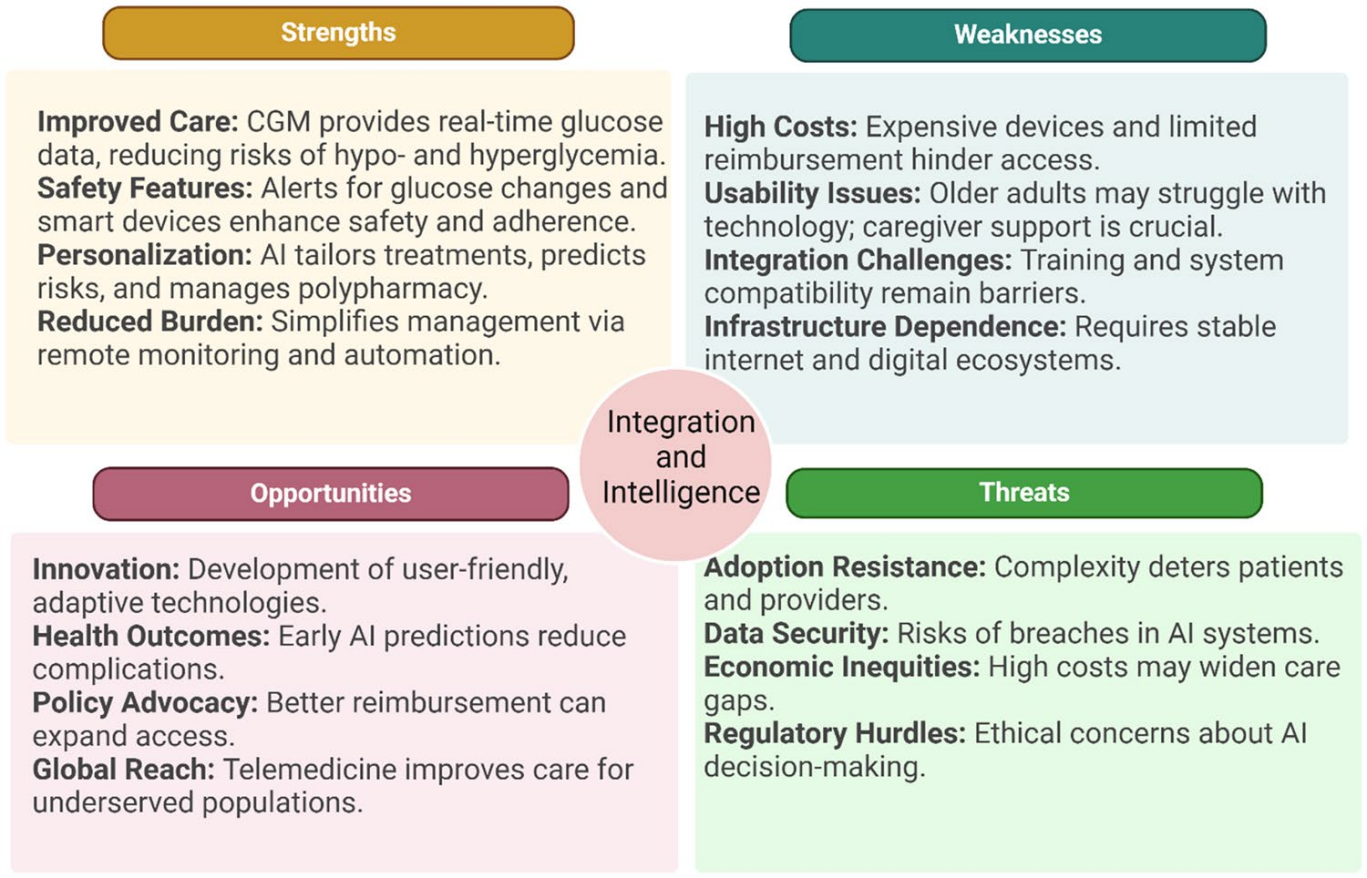
SWOT analysis (Strengths, Weaknesses, Opportunities, Threats) in technology and AI in diabetes management for frail populations. *CGM* continuous glucose monitoring, *AI* artificial intelligence. (Created with BioRender)

Advances in technology and artificial intelligence (AI) are revolutionizing diabetes management, particularly for frail populations who often face unique challenges in disease control. Figure 5 shows an SWOT analysis on technology integration and AI in diabetes management for frail populations. New technologies, ranging from continuous glucose monitoring (CGM) systems [88, 89] to AI-driven decision support tools [90–92], have the potential to improve outcomes by enhancing patient monitoring, simplifying disease management, and offering personalized care tailored to the complexities of older vulnerable patients. These innovations are reshaping the field of diabetes care, especially in terms of improving safety, reducing the burden on patients and caregivers, and optimizing clinical decision-making. However, the potential limitations of these innovations need to be mentioned, including high costs, limited familiarity among older individuals, challenges with reimbursement—both for patients and healthcare providers—and potential barriers to widespread adoption due to the complexity of integrating these therapies into routine care.

CGM technology has become a vital tool in diabetes management, particularly for older adults who may struggle with traditional self-monitoring of blood glucose (SMBG) methods [93]. CGM devices provide real-time glucose data, offering significant advantages in controlling blood glucose levels, especially for patients at risk of hypoglycaemia. CGM devices continuously track glucose levels, allowing for more precise adjustments to medications or lifestyle interventions [94, 95]. Frail older adults are especially vulnerable to hypoglycaemia, which can lead to falls, fractures, and hospitalizations. CGM systems provide alarms for rapidly falling glucose levels, enabling timely interventions to prevent severe hypoglycaemia. This safety feature is particularly valuable for individuals living alone or those with cognitive impairment, reducing the risk of dangerous hypoglycaemic episodes. CGM systems eliminate the need for frequent finger-stick testing, making diabetes management less burdensome for older patients and their caregivers. The data collected by CGM devices can also be shared remotely with healthcare providers, facilitating better communication and timely adjustments to treatment. With the integration of CGM into telehealth platforms, healthcare providers can remotely monitor patients’ glucose trends and intervene promptly when necessary. This can reduce the likelihood of hospitalizations due to uncontrolled diabetes or complications such as infections or cardiovascular events [96]. Insulin pumps complement CGM by delivering insulin continuously, removing the necessity for multiple daily injections. Modern pumps can pair with CGM to automatically adjust insulin doses based on glucose levels, with automated insulin delivery systems facilitating rapid bolus adjustments for meals. Digital diaries and smartphone apps play a crucial role in logging blood glucose levels, food intake, insulin doses, and physical activity. These tools support treatment optimization and enable data sharing with healthcare providers for improved diabetes management. Smart assistants, such as Amazon Echo or Google Home, aid daily life by providing medication and appointment reminders, answering questions, and controlling smart home devices. Wearables like smartwatches track physical activity, provide medication reminders, measure heart rate, detect rhythm disorders, and even call for help in emergencies. Telemedicine and online consultation platforms bring medical advice and care directly to patients, particularly those with mobility challenges or living in rural areas [96]. Emergency alert systems, including wearable buttons or watches with automatic fall detection, ensure quick assistance in emergencies, while smart home technologies like intelligent lighting, thermostats, and security systems enhance safety and convenience through voice commands or smartphone apps. Emerging technologies such as robotic assistance systems are beginning to support household tasks and hold potential for diabetes self-management and reducing loneliness. Electric mobility aids, including wheelchairs, further promote outdoor independence. Together, these digital solutions empower older adults to lead more independent, connected, and health-conscious lives [97]. Diabetes technology holds significant potential for improving the management of frail older adults with diabetes. These advancements can enhance glycaemic control, reduce the burden of frequent clinic visits, and provide timely interventions tailored to individual needs. Telemedicine facilitates continuous monitoring and real-time support, making it an invaluable tool for patients with mobility limitations or those residing in remote areas. By leveraging these technologies, healthcare providers can offer more personalized and accessible care, ultimately improving health outcomes and quality of life in this vulnerable population.

AI is increasingly being used in diabetes management to assist clinicians in making data-driven decisions. These AI-driven systems can support clinicians in managing the complexities of diabetes in frail older patients, offering more tailored and responsive care [95]. AI can create personalized treatment plans based on the patient’s specific needs. This individualized approach ensures that persons receive treatments optimized for their unique physiological and social circumstances. AI systems can analyze historical patient data to predict the likelihood of diabetes-related complications, such as hypoglycaemia, heart disease, or nephropathy. By anticipating these risks, healthcare providers can implement preventive strategies earlier, improving outcomes for older adults who are at heightened risk. AI can help manage polypharmacy in older adults by suggesting the most appropriate diabetes medications while minimizing drug interactions and adverse effects. AI-based tools are particularly useful in balancing glycaemic control with the risk of hypoglycaemia or other complications in frail patients.

Indeed, while the introduction of new technologies and AI into diabetes management offers considerable benefits, there are also challenges, particularly when applied to older and frail populations. Many older adults may struggle with using advanced technology, such as CGM systems or AI-driven apps, due to limited technological literacy. Simplified interfaces and caregiver support are essential to ensure that these technologies can be effectively used by frail populations.

## Conclusions, key messages, and open questions

The integration of frailty into diabetes management is crucial, as it necessitates individualized strategies that go beyond standard protocols. Recent guidelines emphasize simplifying and relaxing treatment regimens, particularly insulin therapy, while aligning glycemic targets with frailty subcategories and life expectancy. Recognizing the metabolic diversity within this population, two distinct phenotypes—anorexic malnourished and sarcopenic obese—have been identified. Each requires tailored management strategies: malnourished individuals benefit from relaxed glycaemic targets and treatment de-intensification, while those with sarcopenic obesity may require tighter glycaemic control combined to address excess fat and preserve muscle mass. Innovative pharmacological agents and digital tools like CGM hold promise, but barriers such as limited technological literacy must be addressed, especially for underserved populations. Multidisciplinary collaboration, caregiver empowerment, and community-based interventions are essential to improve outcomes, particularly for socially deprived or resource-limited individuals. Simplifying treatment regimens, addressing polypharmacy, and prioritizing quality of life while managing cardiovascular risk further enhance patient-centered care. Future research should focus on the long-term safety of new therapies, equitable access to technologies, and practical strategies to integrate frailty into routine diabetes care, optimizing outcomes for this vulnerable population. Table 3 highlights critical open questions across key research areas related to diabetes and frailty in older adults. These questions aim to guide future research and improve outcomes for aging populations. Addressing these challenges will be vital in advancing diabetes care for frail populations and improving their overall quality of life.

**Table 3:** Open questions in research on diabetes and frailty in older adults

Research area	Open questions
Pharmacological research	– What are the long-term effects of SGLT2 inhibitors and GLP-1 receptor agonists on frailty and sarcopenia in older adults?
	– Can dual agonists (e.g., tirzepatide) provide significant benefits in frail populations without exacerbating muscle loss?
Glycaemic targets and safety	– What are the optimal HbA1c targets for different degrees of frailty in older adults?
	– How can hypoglycaemia risk in frail patients be better predicted and mitigated?
Technology in diabetes management	– How effective are AI-driven tools and CGM systems in improving outcomes for older adults with limited technological literacy?
	– What strategies can enhance the accessibility and usability of these technologies for frail populations?
Nutrition and frailty	– What are the most effective nutritional interventions to prevent sarcopenia and frailty progression in older adults with diabetes?
	– How does weight loss induced by metformin, SGLT2 inhibitors, GLP-1 receptor agonists or dual agonists affect body composition, especially muscle mass?
Preventive strategies	– What is the role of early frailty screening in improving diabetes outcomes in older adults?
	– How can preventive care programs be optimized for those with multimorbidity and limited mobility?
	– What is the role of vaccinations in preventing not only infectious diseases but also their downstream effects on the progression of diabetes complications?
Psychosocial and caregiver involvement	– How do social determinants of health impact diabetes outcomes in older adults, particularly those who are frail or cognitively impaired?
	– What role can caregivers play in supporting diabetes management, and how can their involvement be optimized?
Healthcare systems and integration	– How can healthcare systems better integrate comprehensive geriatric assessment and interdisciplinary care for older adults with diabetes and frailty?
	– What cost-effective models of care can address the needs of aging populations with diabetes, especially in underserved areas?

*SGLT2* sodium-glucose cotransporter-2 inhibitors, *GLP-1* glucagon-like peptide-1, *HbA1c* hemoglobin A1c, *AI* artificial intelligence, *CGM* continuous glucose monitoring

## Key considerations for managing diabetes in frail older adults

1. Heterogeneity: Older and frail adults with diabetes are a diverse population with varying functional status, cognitive abilities, and life expectancy. Individualized care plans must address these differences to optimize outcomes.
2. Frailty: Frailty heightens the risk of hypoglycaemia, complications, and poor outcomes. Adjusting glycaemic targets (e.g., HbA1c range of 7.5–8.5% for frail individuals) can reduce overtreatment and associated risks.
3. Nutrition: Nutritional assessment is crucial before initiating antidiabetic therapies with weight-loss properties. Such therapies should be avoided in malnourished individuals or those at risk until nutritional status improves.
4. Weight: Weight loss is not a primary goal in older adults, even for those who are overweight or obese, as it may increase risks of malnutrition and sarcopenia. Careful evaluation of risks and benefits is essential, with weight-loss strategies reserved for carefully selected individuals.
5. Deprescribing: In populations with moderate-to-severe frailty, dementia, or those in nursing homes, deprescribing can minimize hypoglycaemia risk and reduce unnecessary treatment burdens.
6. Therapies: Emerging therapies, such as SGLT2 inhibitors, GLP-1 receptor agonists, and dual agonists, offer cardiovascular, renal, and neuroprotective benefits. However, their effects on frailty, muscle mass, and sarcopenia must be carefully monitored. Promising innovations like triple agonists (e.g., Retatrutide) and fat-reducing, muscle-preserving agents (e.g., Bimagrumab) represent a new frontier in diabetes care.
7. Technology: Continuous glucose monitoring (CGM) and AI-based decision support systems improve glycemic control and safety. However, their usability and accessibility must be adapted to the needs of older adults.
8. Prevention: Comprehensive care includes regular screenings for complications, geriatric syndromes, and vaccinations (e.g., influenza, pneumococcal, COVID-19, zoster, and RSV).
9. Quality of life: For frail or terminally ill patients, care should focus on maintaining functional independence, minimizing treatment burdens, and aligning interventions with individual goals and preferences.
10. Future directions: Greater emphasis is needed on integrating frailty assessments into clinical guidelines and everyday practice.
